# Nutritional-C-reactive protein ratio as a prognostic marker in gastric cancer: a multicenter study

**DOI:** 10.3389/fnut.2026.1840225

**Published:** 2026-08-03

**Authors:** Yibo Chen, Fuquan Yang, Changyang Chen, Pengyu Chen, Haitao Wen, Nuo Xu, Shuyao Wang, Changhong Xu, Siyu Lin, Hanping Shi, Hailun Xie

**Affiliations:** 1 Department of Gastrointestinal and Gland Surgery, The First Affiliated Hospital, Guangxi Medical University, Nanning, Guangxi, China; 2 Guangxi Key Laboratory of Enhanced Recovery After Surgery for Gastrointestinal Cancer, Nanning, Guangxi, China

**Keywords:** cachexia, gastric cancer, malnutrition, NCR, overall survival

## Abstract

**Purpose:**

This study aimed to investigate the association of baseline Nutritional-C-reactive Protein Ratio (NCR) with overall survival (OS), severe malnutrition, and cachexia in patients with gastric cancer.

**Methods:**

Baseline NCR was calculated as BMI (kg/m^2^) × albumin (g/L) / C-reactive protein (mg/L) using measurements obtained at study enrollment and original continuous CRP values. The ROC-derived cutoff was used to classify patients into low- and high-NCR groups. Kaplan–Meier curves and log-rank tests were used to compare OS. Cox proportional hazards models assessed the association between NCR and OS, while logistic regression evaluated associations with severe malnutrition and cachexia.

**Results:**

A total of 1,058 patients with pathologically confirmed gastric cancer were included. The exploratory ROC-derived cutoff value for NCR was 178. Patients with high NCR had significantly better OS than those with low NCR (65.3% vs. 48.4%, log-rank *p* < 0.001). In multivariable Cox regression, high NCR was independently associated with better OS (HR = 0.598, 95% CI: 0.478–0.747, *p* < 0.001), with a significant dose–response relationship across NCR quartiles (p for trend < 0.001). Logistic regression showed that high NCR was associated with lower odds of severe malnutrition (OR = 0.390, 95% CI: 0.300–0.505, *p* < 0.001) and cachexia (OR = 0.182, 95% CI: 0.137–0.242, *p* < 0.001).

**Conclusion:**

Baseline NCR is a simple and clinically accessible composite biomarker integrating nutritional and inflammatory status. It may serve as a complementary marker for risk stratification in patients with gastric cancer.

## Introduction

Gastric cancer (GC) represents a major global public health challenge, ranking fifth in incidence and fourth in cancer-related mortality. In 2020, approximately 1.09 million new cases and 769,000 deaths were recorded, with East Asia accounting for over 50% of both figures, reflecting the region’s heightened susceptibility to this malignancy (1, 2). In China, GC remains a critical public health issue, with incidence and mortality rates ranking fifth and third among all cancers, respectively, both significantly surpassing global averages (3, 4). This substantial disease burden highlights the urgent need for strategies to improve GC prognosis and clinical management.

While conventional prognostic indicators, such as TNM stage and pathological subtype, offer fundamental guidance for clinical decision-making, they fail to account for the complex interplay between systemic inflammation, nutritional status, and tumor biology—three interrelated factors that critically influence treatment tolerance, complication risk, and long-term survival (5–7). Systemic inflammation is a hallmark of advanced GC, driving tumor progression, angiogenesis, and immunosuppression through the release of pro-inflammatory cytokines (8–10). Nutritional decline, including severe malnutrition and cancer cachexia, affects over 50% of GC patients and independently predicts higher treatment-related complication rates and poorer survival (11). These limitations of traditional prognostic tools highlight the need for a simple, non-invasive, and comprehensive biomarker that integrates both inflammatory and nutritional status to improve prognostic accuracy in GC.

The Nutritional-C-reactive Protein Ratio (NCR) is a novel index that integrates nutritional and inflammatory status, derived from routine biochemical and nutritional assessments. It quantifies the balance between nutrition and systemic inflammation by incorporating three key components: body mass index (BMI), serum albumin (ALB)—a fundamental marker of nutritional status—and C-reactive protein (CRP), a well-established marker of systemic inflammation. Inflammation plays a central role in the prognosis of GC, with chronic inflammatory responses in the tumor microenvironment contributing to tumor initiation, progression, invasion, and chemoresistance, ultimately leading to poorer clinical outcomes and reduced long-term survival (12). CRP has consistently been identified as an independent predictor of poor prognosis in patients with GC (13, 14). Nutritional status is another critical determinant of GC prognosis. Malnutrition is prevalent among GC patients and is closely linked to diminished treatment tolerance, increased postoperative complications, accelerated disease progression, and worsened survival (11–15). Among nutritional markers, both serum ALB and BMI are robust indicators of nutritional reserve and predictors of clinical outcome. Given the interplay between inflammation and malnutrition in driving GC progression, several composite indices, such as the lymphocyte-to-CRP ratio (LCR) and the CRP-to-ALB ratio (CAR), have been shown to correlate strongly with prognosis in GC patients, as they reflect multiple pathophysiological processes involved in tumor development (16, 17).

NCR has emerged as a prognostic indicator in colon cancer (18) and has been utilized as a biomarker for assessing nutritional and inflammatory status in cancer cachexia, with predictive value for clinical outcomes (19). However, the role of NCR in GC remains underexplored, particularly in relation to clinically relevant nutrition-related outcomes in this context. This gap is significant given that GC patients are highly susceptible to nutritional deterioration, which directly impacts treatment response and long-term survival. To address this, a multicenter retrospective cohort study involving Chinese GC patients was conducted. This study aimed to evaluate NCR as a tool for identifying malnutrition and cachexia in this cohort and explore its correlation with overall survival (OS). These findings may provide exploratory evidence for supplementary risk stratification in GC, although their clinical applicability requires further validation in independent prospective cohorts.

## Materials and methods

### Study population

The patients included in this study were part of the Investigation on Nutrition Status and its Clinical Outcome of Common Cancers (INSCOC) project, which recruited participants from multiple clinical centers across China starting in 2013. Eligible participants were required to meet the following criteria: aged 18–80 years, pathologically confirmed GC, written informed consent provided, and complete clinical records available. Exclusion criteria included: (a) patients with multiple types of malignant tumors, (b) patients with evident systemic infections or inflammatory symptoms, and (c) patients with severe comorbidities such as heart failure or renal failure. The study protocol was approved by the Institutional Review Board of The First Affiliated Hospital of Guangxi Medical University, and informed consent was obtained from all participants. Patient data were anonymized and de-identified to ensure confidentiality, with all procedures conducted in accordance with the principles of the Declaration of Helsinki.

### Data collection

Data were extracted from electronic medical records, including patient demographics (age, gender), smoking history (current/former smokers *vs.* never smokers), drinking history (current/former drinkers *vs.* never drinkers), family history of cancer, TNM staging (based on the 8th edition of the American Joint Committee on Cancer (AJCC) criteria), anti-tumor treatments (surgery, radiotherapy, chemotherapy), and comorbidities (hypertension, diabetes). Additionally, serum ALB levels (measured using the bromocresol green method, reference range: 35–50 g/L) and CRP levels (measured via immunoturbidimetry, reference range: 0–10 mg/L) were recorded. The BMI, serum albumin, and CRP values used to calculate NCR were baseline measurements obtained at study enrollment. Hypertension was diagnosed according to the 2018 Chinese Guidelines for the Management of Hypertension: systolic blood pressure ≥ 140 mmHg and/or diastolic blood pressure ≥ 90 mmHg, or a previous diagnosis with regular medication. Diabetes was diagnosed based on the World Health Organization (WHO) criteria: fasting blood glucose ≥ 7.0 mmol/L, glycated hemoglobin (HbA1c) ≥ 6.5%, or a prior diagnosis with anti-diabetic treatment. Only patients with type 2 diabetes were included in this study. The missing rate for key variables (BMI, ALB, and CRP) was < 3%, and a complete case analysis was conducted.

### Follow-up

All enrolled patients were prospectively followed up to monitor survival outcomes. The follow-up period started from the date of initial diagnosis (or enrollment) and ended on the cutoff date for data analysis or the date of death, whichever occurred first. Follow-up was primarily conducted through two methods to ensure data accuracy: outpatient re-examinations (including physical exams, imaging tests, and laboratory assessments) and telephone follow-ups (for patients unable to attend regular outpatient visits, with contact made with patients or their family members). Follow-up frequency was adjusted based on clinical guidelines: every 3 months in the first 2 years after diagnosis, every 6 months in years 3–5, and annually thereafter, unless clinical deterioration necessitated more frequent monitoring. The primary outcome of this study was OS, defined as the time from initial diagnosis (or enrollment) to death from any cause.

### Definition of malnutrition

Malnutrition was assessed using the Patient-Generated Subjective Global Assessment (PG-SGA), a validated tool for chronic disease populations that integrates patient-reported data (e.g., weight change, food intake, eating symptoms) with clinician evaluations (e.g., muscle/fat status). Following standardized criteria and prior validation, severe malnutrition was defined as a total PG-SGA score > 8, which indicates significant nutritional deficits typically requiring urgent intervention (18).

### Definition of Cachexia

Cachexia was diagnosed based on the 2023 Asian Cachexia Working Group (AWGC) criteria, with individuals meeting the following conditions: ① Weight loss: > 5% loss within 6 months or > 2% loss when BMI < 20 kg/m^2^; ② Concurrently fulfilling at least one of the following: sarcopenia (assessed by bioelectrical impedance analysis, defined as skeletal muscle index < 7.0 kg/m^2^ in men and < 5.7 kg/m^2^ in women), anorexia, or elevated inflammatory markers (e.g., CRP > 5 mg/L) (19, 20).

### Statistical analysis

Statistical analyses were conducted using IBM SPSS Statistics 26.0 (IBM Corp., Armonk, NY, United States) and R 4.3.2 (R Foundation for Statistical Computing, Vienna, Austria). Continuous variables were expressed as mean ± standard deviation or median (interquartile range), and categorical variables as counts (%). For inter-group comparisons, the t-test or Mann–Whitney U test was used for continuous variables, and the χ^2^ test for categorical variables. The ROC-derived cutoff value of NCR for OS was determined by receiver operating characteristic (ROC) curve analysis by maximizing the Youden index (sensitivity + specificity – 1). Because the discriminatory performance was modest, this cutoff was used as an exploratory threshold for risk stratification within the present cohort. Kaplan–Meier curves were constructed to compare survival between high and low NCR groups, with significance of differences assessed using the log-rank test. Cox proportional hazards regression models were used to assess the relationship between NCR and survival, reporting hazard ratios (HRs) with 95% confidence intervals (CIs). Three models were built: Model a (unadjusted), Model b (adjusted for age, gender, and TNM stage), and Model c (further adjusted for surgery, radiotherapy, chemotherapy, hypertension, diabetes, smoking, drinking, and family history of cancer). The non-linear relationship between NCR and OS in GC patients was evaluated using restricted cubic splines (RCS). Logistic regression models assessed the association between NCR and severe malnutrition or cachexia, reporting odds ratios (ORs) with 95% CIs. Sensitivity analysis was performed by excluding patients who died within 3 months of diagnosis to mitigate early-death bias. All tests were two-sided, with *p* < 0.05 considered statistically significant.

## Results

### Patient characteristics

A total of 1,058 GC patients were enrolled in this study, with 70.9% male and a mean age of 59.49 ± 11.67 years. Among them, 68.6% were diagnosed with TNM stage III–IV disease. ROC curve analysis identified an exploratory NCR cutoff value of 178 for OS, with modest discriminatory performance (AUC = 0.586; sensitivity = 57.6%; specificity = 59.2%) (Supplementary Figure S1). Based on this cutoff, patients were classified into two groups: low NCR (< 178, *n* = 510) and high NCR (≥ 178, *n* = 548). The low NCR group exhibited significantly poorer clinical and laboratory characteristics compared to the high NCR group. The mean age was higher in the low NCR group (60.70 years) compared to the high NCR group (58.37 years). The low NCR group also had higher prevalence rates of hypertension, diabetes, smoking, and alcohol consumption. A greater proportion of patients in the low NCR group presented with TNM stage III–IV disease. Laboratory results indicated that the low NCR group had a higher median neutrophil count, lower median lymphocyte count, lower median ALB level, and higher median CRP level. Furthermore, patients in the low NCR group had higher PG-SGA scores, reflecting a higher incidence of severe malnutrition. Rates of cancer cachexia and overall mortality were also significantly higher in the low NCR group. All between-group differences were statistically significant, as detailed in Supplementary Table S1.

### Kaplan–Meier survival analysis of NCR

**Figure 1:**
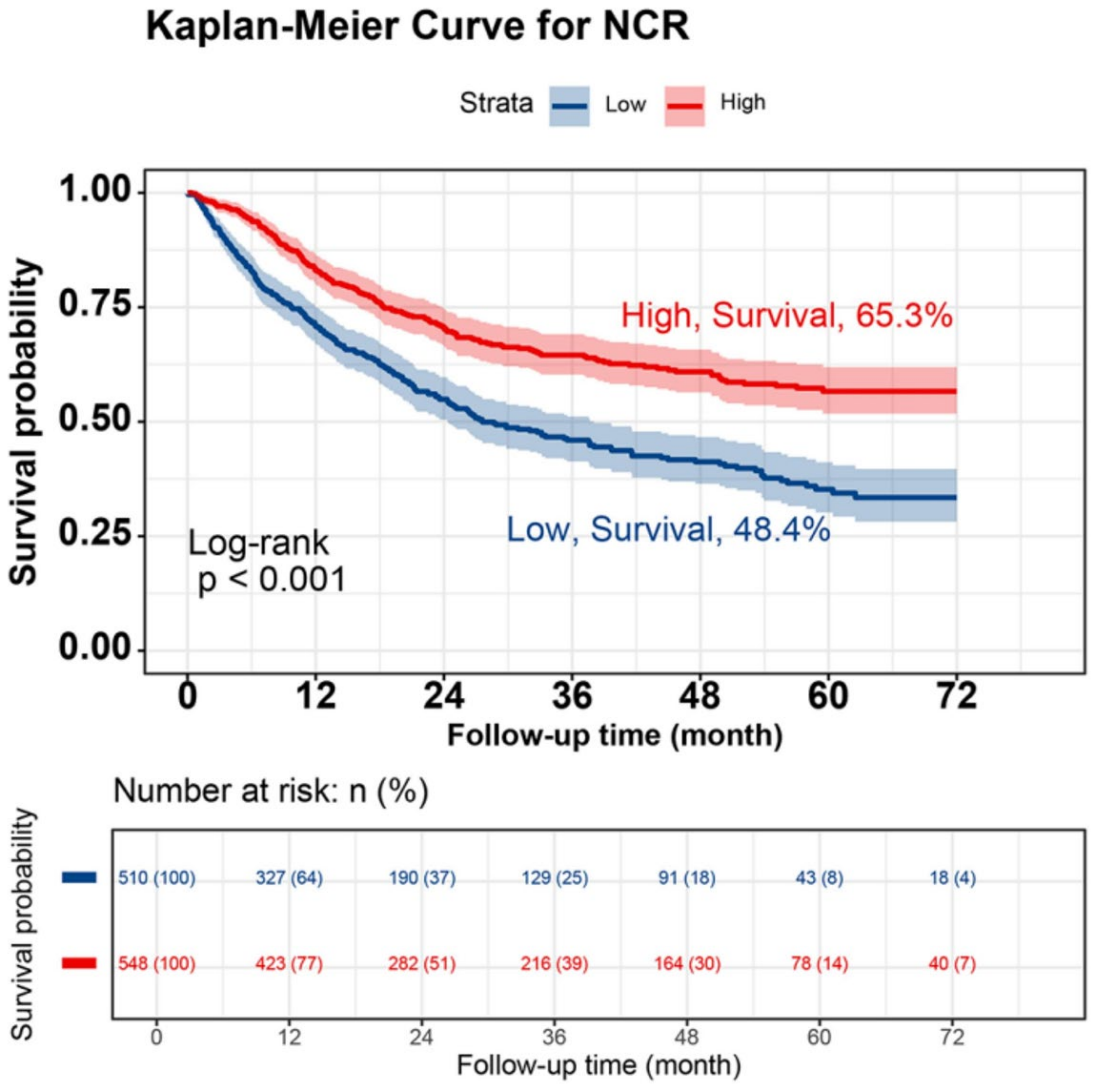
Kaplan–Meier curve of NCR in patients with gastric cancer. Kaplan–Meier survival curves according to baseline NCR group in patients with gastric cancer. Patients were classified into low- and high-NCR groups using the exploratory ROC-derived cutoff of 178. Survival differences were assessed using the log-rank test, and numbers at risk are shown below the curves.

The median follow-up time was 21 (1–72) months. During this period, 453 (42.8%) patients died. The crude mortality rate was higher in the low-NCR group than in the high-NCR group (51.6% vs. 34.7%; *p* < 0.001). Kaplan–Meier analysis showed that patients in the high-NCR group had significantly better OS than those in the low-NCR group, with survival rates of 65.3 and 48.4%, respectively (log-rank test, *p* < 0.001) (Figure 1). Subgroup analysis stratified by TNM stage further validated the prognostic role of NCR across different disease stages. In patients with TNM stage I–II disease, the high NCR group showed a numerically higher survival rate of 79.2% compared to 71.9% in the low NCR group. Although the high NCR group exhibited a more favorable survival trend, the difference did not reach statistical significance (*p* = 0.06) (Supplementary Figure S2A). Among patients with stage III–IV disease, the high NCR group demonstrated a significantly higher survival rate than the low NCR group, with survival rates of 57.5% versus 40.0%, respectively, and this difference was statistically significant (*p* < 0.001) (Supplementary Figure S2B).

### Cox proportional hazards regression analysis for the prognostic role of NCR

**Figure 2:**
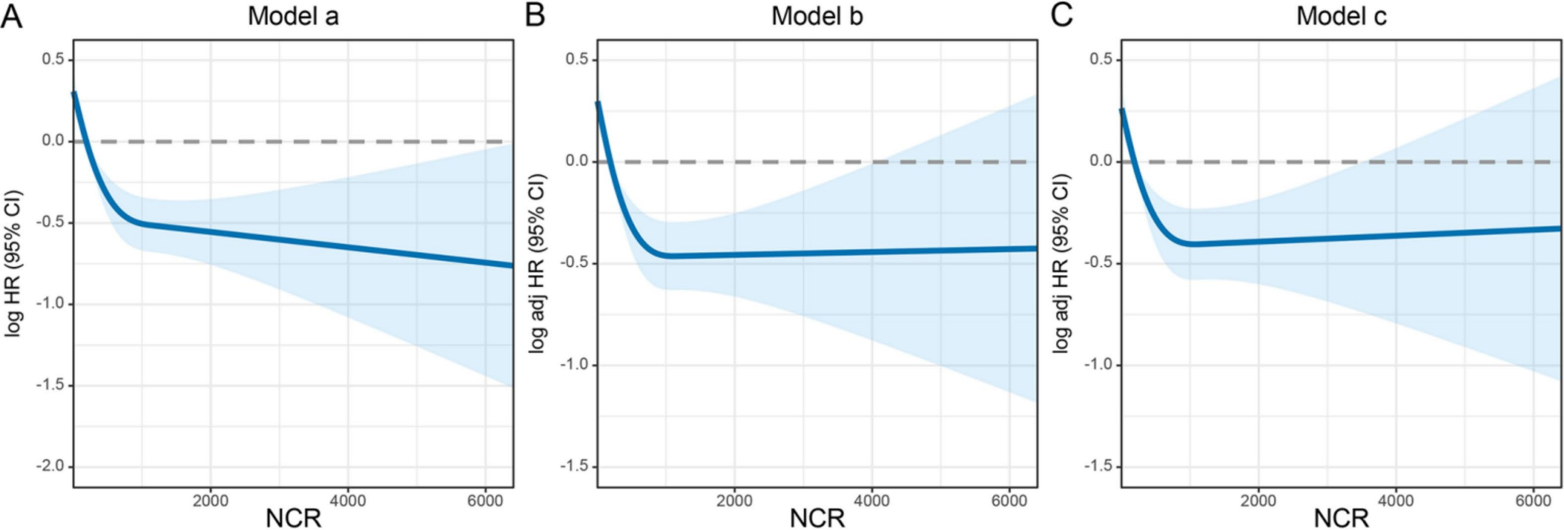
Association of NCR with all cause mortality in patients with gastric cancer. **(A)** Model a: no adjusted. **(B)** Model b: adjusted for age, sex, TNM stage. **(C)** Model c: adjusted for age, sex, TNM stage, surgery, radiotherapy, chemotherapy, hypertension, diabetes, smoking, drinking, family history.

**Figure 3:**
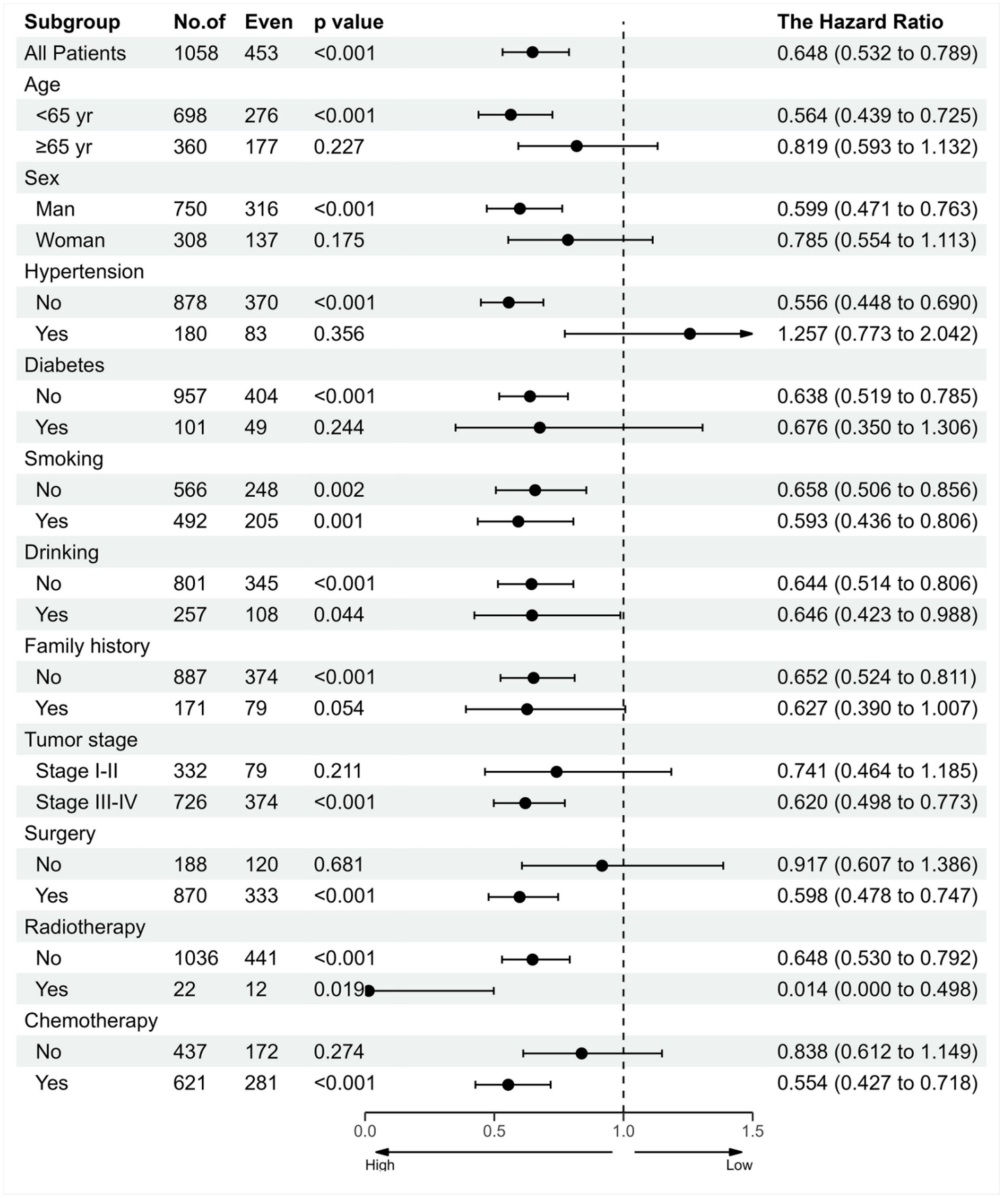
Subgroup survival forest plot. Subgroup forest plot for the association between high NCR and overall survival. Hazard ratios below 1 indicate lower mortality risk in the high-NCR group. Subgroup findings should be interpreted cautiously because event numbers varied across strata.

In multivariate Cox proportional hazards regression analysis, when NCR was treated as a continuous variable, every one standard deviation increase in NCR was associated with a 10.7% reduction in the risk of poor prognosis for GC patients (HR = 0.893, 95% CI = 0.774–0.981, *p* = 0.033). High NCR remained significantly associated with lower all-cause mortality across all models: HR = 0.544 (95% CI: 0.451–0.656, *p* < 0.001) in Model a; HR = 0.601 (95% CI: 0.497–0.726, *p* < 0.001) in Model b; and HR = 0.598 (95% CI: 0.478–0.747, *p* < 0.001) in Model c. When NCR was divided into quartiles, higher NCR quartiles, particularly Q3 and Q4, were associated with lower all-cause mortality, with the lowest quartile as the reference. After adjusting for confounders, the HRs for all-cause mortality were 0.889 (95% CI: 0.659–1.199) for Q2, 0.620 (95% CI: 0.452–0.850) for Q3, and 0.507 (95% CI: 0.373–0.691) for Q4 (Table 1). The RCS analysis revealed a consistent negative dose–response relationship between NCR and the risk of adverse prognosis in GC patients across all adjusted models (Figure 2). These results suggested that incremental increases in NCR were associated with corresponding improvements in prognosis. Sensitivity analysis excluding 58 patients who died within 3 months of diagnosis showed a consistent association between high NCR and better OS. In the fully adjusted model (Model c), high NCR levels remained significantly associated with better OS, with an HR of 0.699 (95% CI: 0.566–0.863, *p* = 0.001). The significant dose–response relationship across NCR quartiles persisted, with a p for trend < 0.001 (Supplementary Table S2). In subgroup analysis, after adjusting for confounding factors, NCR was identified as an independent prognostic factor in 14 of the 22 subgroups (Figure 3).

**Table 1:** Association between NCR and survival of patients with gastric cancer.

NCR	Model a	*p* value	Model b	*p* value	Model c	*p* value
Continuous (per SD)	0.8 (0.696,0.92)	0.002	0.847 (0.736,0.975)	0.021	0.893 (0.774, 0.981)	0.033
Cutoff value						
C1 (<178)	Ref		Ref		Ref	
C2 (≥178)	0.544 (0.451,0.656)	<0.001	0.601 (0.497,0.726)	<0.001	0.598 (0.478,0.747)	<0.001
Quartiles						
Q1 (<39.52)	Ref		Ref		Ref	
Q2 (39.52–190.72)	1.165 (0.917,1.48)	0.21	0.953 (0.749,1.214)	0.698	0.889 (0.659,1.199)	0.442
Q3 (190.72–417.22)	0.714 (0.548,0.929)	0.012	0.678 (0.521,0.884)	0.004	0.62 (0.452,0.85)	0.003
Q4 (≥417.22)	0.485 (0.37,0.636)	<0.001	0.503 (0.383,0.659)	<0.001	0.507 (0.373,0.691)	<0.001
p for trend		<0.001		<0.001		<0.001

Model a: No adjusted. Model b: Adjusted for age, sex, TNM stage. Model c: Adjusted for age, sex, TNM stage, surgery, radiotherapy, chemotherapy, hypertension, diabetes, smoking, drinking, family history.

### Logistic regression analysis of the relationship between NCR and severe malnutrition

A total of 515 (48.7%) participants developed severe malnutrition. Compared to patients in the high NCR group, those in the low NCR group exhibited significantly higher PG-SGA scores, with median values of 9.00 (interquartile range, 6.00–12.00) versus 6.00 (interquartile range, 3.00–9.00; *p* < 0.001). When NCR was analyzed as a continuous variable, each one standard deviation increase in NCR was associated with a 36.3% reduction in the risk of severe malnutrition (OR = 0.637, 95% CI = 0.514–0.789; *p* < 0.001). Logistic regression further confirmed that high NCR levels were inversely associated with severe malnutrition (OR = 0.390, 95% CI: 0.300–0.505; *p* < 0.001). When NCR was categorized into quartiles with the lowest quartile (Q1) as the reference, higher NCR quartiles, particularly Q3 and Q4, were associated with lower odds of severe malnutrition. In the fully adjusted model, Q3 (OR = 0.418, 95% CI: 0.290–0.602, *p* < 0.001) and Q4 (OR = 0.267, 95% CI: 0.184–0.387, *p* < 0.001) were significantly associated with lower odds of severe malnutrition, whereas Q2 showed a borderline but non-significant association (OR = 0.706, 95% CI: 0.492–1.013, *p* = 0.059). The trend across quartiles remained significant (p for trend < 0.001) (Table 2).

**Table 2:** Association between NCR and severe malnutrition (PG-SGA>8) of patients with gastric cancer.

NCR	Model a	*p* value	Model b	*p* value	Model c	*p* value
Continuous (per SD)	0.621 (0.504,0.766)	<0.001	0.64 (0.518,0.791)	<0.001	0.637 (0.514,0.789)	<0.001
Cutoff value		<0.001		<0.001		<0.001
C1 (<178)	Ref		Ref		Ref	
C2 (≥178)	0.392 (0.306,0.502)		0.415 (0.323,0.534)		0.39 (0.3,0.505)	
Quartiles						
Q1 (<39.52)	Ref		Ref		Ref	
Q2 (39.52–190.72)	0.772 (0.545,1.094)	0.146	0.721 (0.506,1.028)	0.071	0.706 (0.492,1.013)	0.059
Q3 (190.72–417.22)	0.453 (0.32,0.642)	<0.001	0.454 (0.319,0.646)	<0.001	0.418 (0.290,0.602)	<0.001
Q4 (≥417.22)	0.268 (0.187,0.384)	<0.001	0.281 (0.195,0.404)	<0.001	0.267 (0.184,0.387)	<0.001
p for trend		<0.001		<0.001		<0.001

Model a: No adjusted. Model b: Adjusted for age, sex, TNM stage. Model c: Adjusted for age, sex, TNM stage, surgery, radiotherapy, chemotherapy, hypertension, diabetes, smoking, drinking, family history.

### Logistic regression analysis of the relationship between NCR and Cachexia

A total of 633 (59.8%) patients developed cancer cachexia. Compared to those in the high NCR group, patients in the low NCR group had a significantly higher prevalence of cachexia (79.4% vs. 41.6%, *p* < 0.001). High NCR levels were strongly inversely associated with the risk of cachexia. After adjusting for all covariates, each one standard deviation increase in NCR was associated with lower odds of cachexia (OR = 0.786, 95% CI = 0.676–0.913, *p* = 0.002). In the fully adjusted model (Model c), a high NCR level (≥ 178) was associated with a significantly lower risk of cachexia compared to a low NCR level, with an OR of 0.182 (95% CI = 0.137–0.242, *p* < 0.001). When NCR was divided into quartiles, compared with the lowest quartile (Q1), Q2, Q3, and Q4 were associated with lower odds of cachexia, with ORs of 0.566 (95% CI: 0.368–0.870, *p* = 0.010), 0.125 (95% CI: 0.082–0.190, *p* < 0.001), and 0.161 (95% CI: 0.107–0.243, *p* < 0.001), respectively. The trend was highly significant (p for trend < 0.001) (Table 3).

**Table 3:** Association between NCR and AWGC-defined cachexia of patients with gastric cancer.

NCR	Model a	*p* value	Model b	*p* value	Model c	*p* value
Continuous (per SD)	0.763 (0.658, 0.883)	<0.001	0.782 (0.675, 0.907)	0.001	0.786 (0.676, 0.913)	0.002
Cutoff value		<0.001		<0.001		<0.001
C1 (<178)	Ref		Ref		Ref	
C2 (≥178)	0.185 (0.14, 0.243)		0.188 (0.142, 0.248)		0.182 (0.137, 0.242)	
Quartiles						
Q1 (<39.52)	Ref		Ref		Ref	
Q2 (39.52–190.72)	0.605 (0.397, 0.922)	0.019	0.568 (0.371, 0.869)	0.009	0.566 (0.368, 0.870)	0.010
Q3 (190.72–417.22)	0.134 (0.09, 0.201)	<0.001	0.128 (0.085, 0.193)	<0.001	0.125 (0.082, 0.190)	<0.001
Q4 (≥417.22)	0.161 (0.108, 0.24)	<0.001	0.165 (0.110, 0.247)	<0.001	0.161 (0.107, 0.243)	<0.001
p for trend		<0.001		<0.001		<0.001

Model a: No adjusted. Model b: Adjusted for age, sex, TNM stage. Model c: Adjusted for age, sex, TNM stage, surgery, radiotherapy, chemotherapy, hypertension, diabetes, smoking, drinking, family history.

## Discussion

In recent years, the prognostic significance of systemic inflammatory and nutritional status in malignant tumors has emerged as a key research focus. Composite biomarkers that combine individual inflammatory or nutritional indices have been well validated for their prognostic relevance in GC patients (20, 21). However, these biomarkers typically assess only one dimension—either inflammation or nutrition—failing to capture the synergistic and interactive effects of these two interconnected factors on GC progression. The NCR is a newly developed nutritional–inflammatory composite index that quantifies the balance between nutritional reserve and systemic inflammatory burden. This multicenter retrospective cohort study systematically validated the prognostic value of NCR in a large cohort of Chinese GC patients and, for the first time, explored its specific associations with GC-related nutrition-related adverse outcomes, such as severe malnutrition and cancer cachexia. Consistent with prior studies on NCR’s prognostic role in colorectal cancer and pan-cancer cachectic cohorts (18, 19), our findings confirm that NCR is an independent protective factor for OS in GC patients. Notably, several novel GC-specific insights that extend the clinical applicability of NCR were identified, addressing critical gaps in NCR-related research for GC and providing an additional tool for more refined prognostic assessment and individualized clinical management of GC patients. To further clarify the clinical positioning of NCR, we added a concise comparison of NCR with established inflammation- and nutrition-based prognostic indices, including GPS, mGPS, PNI, NLR, SII, CAR, and LCR, in Supplementary Table S3.

From our perspective, a low NCR may serve as a composite indicator of host inflammatory and nutritional stress. CRP is a classical acute-phase reactant that reflects systemic inflammatory activity, which may accompany tumor progression and cachexia-related metabolic disturbances (22). Sustained inflammation can promote catabolism and nutritional depletion. Accordingly, lower BMI and albumin levels in patients with cachexia may indicate limited anabolic reserve, impaired treatment tolerance, and an established wasting phenotype, consistent with previous NCR-related findings in multicancer cachexia (19). The relationship between inflammation and malnutrition in cancer cachexia may not be fully explained by a simple linear model. Circulating biomarkers may also reflect underlying biological alterations at the tumor–host interface. Macrophage-dominant immune-metabolic programs in the tumor microenvironment can reshape cytokine signaling, substrate utilization, and redox homeostasis, thereby contributing to systemic metabolic imbalance and progressive wasting (23). Mitochondrial dysfunction may provide another relevant mechanistic dimension. In gastric carcinoma, disturbed mitochondrial homeostasis has been linked to oxidative stress, metabolic reprogramming, reduced apoptotic responsiveness, treatment resistance, and tumor progression (24, 25). Experimental evidence also suggests that PINK1-related mitochondrial vulnerability may influence mitochondria-mediated cell death in gastric adenocarcinoma (26, 27). Together, these observations support the interpretation that low NCR may reflect a clinically meaningful phenotype involving inflammatory burden, nutritional depletion, immune-metabolic remodeling, and mitochondrial stress.

A key novel finding of this study is the stage-specific prognostic value of NCR in GC. Subgroup analysis stratified by TNM stage revealed that the survival benefit of a high NCR was statistically significant only in patients with stage III–IV GC. In stage I–II patients, a favorable survival trend was observed, but it did not reach statistical significance. This discrepancy likely reflects the distinct disease characteristics across stages: early-stage GC patients experience milder systemic inflammatory responses and less pronounced nutritional decline, so the heterogeneity in nutritional and inflammatory status is insufficient to produce a statistically significant survival difference. In contrast, advanced GC patients are more prone to pronounced tumor-related inflammation and rapid nutritional depletion. As an integrated index of both nutrition and inflammation, NCR more effectively captures the pathophysiological heterogeneity in this group, offering stronger prognostic stratification. These results suggest that NCR is a stage-adapted prognostic biomarker for GC, with greater clinical utility in advanced disease, where it can complement the TNM staging system for more precise risk stratification.

This study observed that NCR was more strongly associated with cachexia than with severe malnutrition in patients with GC. However, this finding should be interpreted cautiously because CRP is included in both the NCR formula and the cachexia definition. Therefore, the observed association between NCR and cachexia may partly reflect overlapping inflammatory components rather than a fully independent predictive relationship. Cachexia is a complex syndrome driven by interactions between systemic inflammation and dysregulated nutritional metabolism (19, 20), while NCR combines three core indicators—BMI (representing body mass and muscle reserve), ALB (indicating systemic nutritional status), and CRP (indicating inflammatory burden). Thus, NCR more comprehensively captures the pathophysiological features of cachexia than a single PG-SGA score for malnutrition assessment. Nevertheless, because NCR includes BMI and albumin, and because cachexia definitions also involve body weight/BMI and inflammatory markers, the observed associations may partly reflect overlapping constructs rather than a fully independent predictive relationship. Therefore, NCR should not be interpreted as a standalone diagnostic marker for malnutrition or cachexia. These results suggest that NCR may be associated with cachexia risk in patients with GC, but further studies are needed to determine whether NCR can improve the early identification or management of cachexia in clinical practice.

The stable prognostic value of NCR was further validated by sensitivity and dose–response analyses. After excluding patients who died within 3 months of diagnosis, the protective effect of a high NCR on OS remained significant, and the dose–response relationship across quartiles remained robust. This indicates that the prognostic value of NCR is not influenced by early-death bias and demonstrates good clinical reproducibility. RCS analysis also revealed a continuous negative dose–response association between NCR and the risk of adverse prognosis, while the quartile analysis showed that the mortality risk in Q4 was nearly 50% lower than in Q1. This linear relationship suggests that increasing NCR corresponds to a gradual improvement in survival prognosis, providing a quantitative basis for clinical use. Accordingly, the cutoff of 178 should be regarded as an exploratory, cohort-derived threshold rather than a fixed clinical decision point; in practice, NCR is more plausibly used as an adjunct to TNM stage, treatment information, and structured nutritional assessment than as a standalone prognostic classifier.

The main clinical advantages of NCR lie in its simplicity, accessibility, and cost-effectiveness. It is calculated using just three routine clinical indicators (BMI, ALB, CRP), which are readily available from preoperative basic examinations, requiring no additional invasive tests or specialized equipment. This makes NCR particularly suited for settings with limited medical resources, such as primary hospitals and grassroots medical institutions, and enhances its potential for large-scale clinical implementation. Future prospective studies should evaluate whether NCR can improve risk stratification in specific treatment decision contexts. At present, NCR should be considered a supplementary marker rather than a tool for selecting adjuvant therapy or guiding individualized treatment decisions. The mechanistic basis of NCR’s prognostic and predictive value lies in its ability to integrate systemic nutritional reserve and inflammatory burden. ALB and BMI are central markers of nutritional status, directly influencing GC patients’ tolerance to tumor-induced catabolism and anti-tumor therapies. In contrast, CRP is a canonical marker of systemic inflammation; tumor-associated chronic inflammation is widely recognized to promote tumor progression, angiogenesis, and immunosuppression—pathological processes that accelerate disease advancement and worsen clinical outcomes (28, 29). By combining these two key pathophysiological dimensions, NCR offers a comprehensive measure of the balance between the host’s anti-tumor defense and tumor-mediated tissue damage. This integrated metric enables robust prediction of both OS and nutrition-related adverse events in GC patients.

This study has several limitations. First, as a retrospective analysis, selection bias and residual confounding cannot be excluded, and the findings do not establish causality. Second, only baseline NCR values were analyzed, and dynamic changes in BMI, albumin, CRP, or NCR during anti-tumor treatment were not evaluated. Third, the ROC-derived cutoff value of 178 showed modest discriminatory performance and was derived from the present cohort; therefore, it should be considered exploratory and requires external validation in independent cohorts. Fourth, because CRP is included in both the NCR formula and the cachexia definition, the association between NCR and cachexia should be interpreted cautiously, as it may partly reflect overlapping inflammatory components rather than a fully independent predictive relationship. Similarly, because NCR incorporates BMI and albumin, its association with severe malnutrition may partly reflect shared nutritional constructs. Fifth, several potentially important confounders, including performance status, detailed tumor characteristics, metastatic burden, and nutritional support, were unavailable or incomplete in the retrospective database and therefore could not be fully adjusted for. In addition, we did not directly measure circulating cytokines, mitochondrial function, ROS-related markers, or other metabolic intermediates; for that reason, the mechanistic interpretation proposed here remains hypothesis-generating rather than confirmatory. Finally, the study did not assess the relationship between NCR and GC molecular subtypes, such as HER2 expression or microsatellite instability, limiting the integration of NCR with molecular prognostic markers.

## Conclusion

In conclusion, NCR is a simple and clinically accessible composite biomarker integrating nutritional and inflammatory status. In this multicenter cohort of patients with gastric cancer, higher baseline NCR was independently associated with better overall survival and lower odds of severe malnutrition and cachexia. NCR may serve as a useful complementary marker for risk stratification, particularly in patients with advanced gastric cancer. Further prospective studies are needed to validate its clinical applicability.

## Data Availability

The original contributions presented in the study are included in the article/Supplementary material, further inquiries can be directed to the corresponding authors.

## References

[1] Sung H Ferlay J Siegel RL Laversanne M Soerjomataram I Jemal A . Global Cancer statistics 2020: GLOBOCAN estimates of incidence and mortality worldwide for 36 cancers in 185 countries. CA Cancer J Clin. (2021) 71:209–49. doi: 10.3322/caac.21660, 33538338

[2] Siegel RL Giaquinto AN Jemal A. Cancer statistics, 2024. CA Cancer J Clin. (2024) 74:12–49. doi: 10.3322/caac.21820, 38230766

[3] Cao M Li H Sun D Chen W. Cancer burden of major cancers in China: a need for sustainable actions. Cancer Commun. (2020) 40:205–10. doi: 10.1002/cac2.12025, 32359212 PMC7667573

[4] Han B Zheng R Zeng H Wang S Sun K Chen R . Cancer incidence and mortality in China, 2022. J Natl Cancer Cent. (2024) 4:47–53. doi: 10.1016/j.jncc.2024.01.006, 39036382 PMC11256708

[5] Chen D Fu M Chi L Lin L Cheng J Xue W . Prognostic and predictive value of a pathomics signature in gastric cancer. Nat Commun. (2022) 13:6903. doi: 10.1038/s41467-022-34703-w, 36371443 PMC9653436

[6] Zhao J Zhang W Lu D Shao C Chen Y Huang X . Clinical prognostic risk assessment of different pathological subtypes of papillary thyroid cancer: a systematic review and network meta-analysis. Langenbeck's Arch Surg. (2025) 410:251. doi: 10.1007/s00423-025-03841-2, 40853492 PMC12378718

[7] Sugawara K Yamashita H Urabe M Uemura Y Okumura Y Yagi K . Combining nutritional status with TNM stage: a physiological update on gastric cancer staging for improving prognostic accuracy in elderly patients. Int J Clin Oncol. (2022) 27:1849–58. doi: 10.1007/s10147-022-02250-5, 36255516

[8] Coussens LM Werb Z. Inflammation and cancer. Nature. (2002) 420:860–7. doi: 10.1038/nature01322, 12490959 PMC2803035

[9] Jaroenlapnopparat A Bhatia K Coban S. Inflammation and gastric cancer. Diseases (Basel). (2022) 10:35. doi: 10.3390/diseases10030035, 35892729 PMC9326573

[10] Choi EL Taheri N Chandra A Hayashi Y. Cellular senescence, inflammation, and Cancer in the gastrointestinal tract. Int J Mol Sci. (2023) 24:9810. doi: 10.3390/ijms24129810, 37372958 PMC10298598

[11] Kang MK Lee HJ. Impact of malnutrition and nutritional support after gastrectomy in patients with gastric cancer. Ann Gastroenterol Surg. (2024) 8:534–52. doi: 10.1002/ags3.12788, 38957563 PMC11216795

[12] Wei X Xie F Zhou X Wu Y Yan H Liu T . Role of pyroptosis in inflammation and cancer. Cell Mol Immunol. (2022) 19:971–92. doi: 10.1038/s41423-022-00905-x, 35970871 PMC9376585

[13] Shen DY Li ZY Xia WB Li D Zhao S Yang X . Long-term prognostic value of postoperative C-reactive protein in gastric cancer patients: a systematic review and meta-analysis. BMC Gastroenterol. (2025) 25:887. doi: 10.1186/s12876-025-04481-y, 41299309 PMC12752049

[14] Lu J Xu BB Xue Z Xie JW Zheng CH Huang CM . Perioperative CRP: a novel inflammation-based classification in gastric cancer for recurrence and chemotherapy benefit. Cancer Med. (2021) 10:34–44. doi: 10.1002/cam4.3514, 33270989 PMC7826470

[15] Zheng J Wang X Yu J Hu Q Zhan Z Zhou S . Global leadership initiative on malnutrition criteria: clinical benefits for patients with gastric cancer. Nutr Clin Pract. (2025) 40:239–51. doi: 10.1002/ncp.11224, 39499024

[16] Miyatani K Sawata S Makinoya M Miyauchi W Shimizu S Shishido Y . Combined analysis of preoperative and postoperative lymphocyte-C-reactive protein ratio precisely predicts outcomes of patients with gastric cancer. BMC Cancer. (2022) 22:641. doi: 10.1186/s12885-022-09716-9, 35690739 PMC9188155

[17] Christodoulidis G Voutyras A Fotakopoulos G Koumarelas KE Georgakopoulou VE Kouliou MN . CRP to albumin ratio as a prognostic nutrition-based biomarker for patients with gastric Cancer: a narrative review. Cureus. (2024) 16:e71516. doi: 10.7759/cureus.71516, 39553069 PMC11563776

[18] Langheinrich M Siebenhüner AR Baecker J Miragall M Wiesmüller F Schellerer V . NCR, an inflammation and nutrition related blood-based marker in colon cancer patients: a new promising biomarker to predict outcome. Diagnostics (Basel). (2022) 13:116. doi: 10.3390/diagnostics13010116, 36611408 PMC9818830

[19] Li X Deng L Xie H Li S Zhao H Liu T . NCR as a biomarker for nutritional status and inflammation in predicting outcomes in patients with cancer cachexia: a prospective, multicenter study. BMC Cancer. (2025) 25:539. doi: 10.1186/s12885-025-13919-1, 40133874 PMC11934689

[20] Yamamoto T Kawada K Obama K. Inflammation-related biomarkers for the prediction of prognosis in colorectal Cancer patients. Int J Mol Sci. (2021) 22:8002. doi: 10.3390/ijms22158002, 34360768 PMC8348168

[21] Okugawa Y Toiyama Y Yamamoto A Shigemori T Ichikawa T Yin C . Lymphocyte-to-C-reactive protein ratio and score are clinically feasible nutrition-inflammation markers of outcome in patients with gastric cancer. Clin Nutr. (2020) 39:1209–17. doi: 10.1016/j.clnu.2019.05.009, 31155370

[22] Ansar W Ghosh S. "Inflammation and inflammatory diseases, markers, and mediators: role of CRP in some inflammatory diseases". In: Ansar W Ghosh S, editors. Biology of C Reactive Protein in Health and Disease. New Delhi: Springer India (2016). p. 67–107.

[23] Dubey S Ghosh S Goswami D Ghatak D De R. Immunometabolic attributes and mitochondria-associated signaling of tumor-associated macrophages in tumor microenvironment modulate cancer progression. Biochem Pharmacol. (2023) 208:115369. doi: 10.1016/j.bcp.2022.115369, 36481347

[24] Arjunan A Venkatraman G Joseph LD Perumalsamy LR. Mitochondrial homeostasis and their impact on gastric carcinoma. Int J Biochem Cell Biol. (2025) 186:106827. doi: 10.1016/j.biocel.2025.106827, 40617267

[25] Hasan S. Y. Awad M. A. Mohsin Z. Humade Z. A. Gawy K. X. Mitochondria in cancer patients: integrating metabolic reprogramming, signaling networks, and therapeutic vulnerabilities. (2026). (Accessed April 12, 2026). Available online at: https://ssrn.com/abstract=6563099

[26] Ghosh S Dutta R Ghatak D Goswami D De R. Immunometabolic characteristics of dendritic cells and its significant modulation by mitochondria-associated signaling in the tumor microenvironment influence cancer progression. Biochem Biophys Res Commun. (2024) 726:150268. doi: 10.1016/j.bbrc.2024.150268, 38909531

[27] Ghosh S Dutta R Goswami D Ghatak D De R. Mitochondrial dynamics and metabolic attributes regulate function of natural killer cell and infiltration in tumor microenvironment modulating disease progression. Biochim Biophys Acta Rev Cancer. (2025) 1880:189471. doi: 10.1016/j.bbcan.2025.189471, 41075850

[28] Tsujiura M Yamamoto A Imaoka H Shimura T Kitajima T Morimoto Y . Clinical utility of lymphocyte to C-reactive protein ratio in predicting survival and postoperative complication for esophago-gastric junction cancer. Surg Oncol. (2022) 44:101842. doi: 10.1016/j.suronc.2022.101842, 36081281

[29] Brown JC Compton SLE Kang A Jayaraman A Gilmore LA Kirby BJ . Effects of exercise on inflammation, circulating tumor cells, and circulating tumor DNA in colorectal cancer. J Sport Health Sci. (2025) 14:101036. doi: 10.1016/j.jshs.2025.101036, 40107449 PMC12341639

