# Supplementary material for: Nutritional-C-reactive protein ratio as a prognostic marker in gastric cancer: a multicenter study
Source: Front Nutr. 2026 Aug 3;13:1840225. doi: 10.3389/fnut.2026.1840225 (PMC13477951; doi:10.3389/fnut.2026.1840225)
Supplement: Supplementary file 1 [file Table_1.docx]

**Figure S1.** The Cut-off of NCR in patients with gastric cancer.

**Figure S2.** Subgroup survival analysis of NCR based on pathological stage.

**Notes：**A, Stage I-II; B, Stage III-IV.

**Table S1.** Characteristics by level of NCR in patients with gastric cancer.

| Characteristic | Overall  (n=1058) | NCR | | |
| --- | --- | --- | --- | --- |
|  |  | Low (n=510) | High (n=548) | p value |
| Sex, male, n (%) | 750 (70.9) | 365 (71.6) | 385 (70.3) | 0.688 |
| Age, years, mean (SD) | 59.49 (11.67) | 60.70 (11.89) | 58.37 (11.36) | 0.001 |
| Hypertension, yes, n (%) | 180 (17.0) | 105 (20.6) | 75 (13.7) | 0.004 |
| Diabetes, yes, n (%) | 101 ( 9.5) | 62 (12.2) | 39 ( 7.1) | 0.007 |
| Smoking yes, n (%) | 492 (46.5) | 259 (50.8) | 233 (42.5) | 0.008 |
| Drinking, yes, n (%) | 257 (24.3) | 144 (28.2) | 113 (20.6) | 0.005 |
| Family history, yes, n (%) | 171 (16.2) | 81 (15.9) | 90 (16.4) | 0.877 |
| TNM stage, Stage III-IV, n (%) | 726 (68.6) | 375 (73.5) | 351 (64.1) | 0.001 |
| Surgery, yes, n (%) | 870 (82.2) | 385 (75.5) | 485 (88.5) | <0.001 |
| Radiotherapy, yes, n (%) | 22 ( 2.1) | 13 ( 2.5) | 9 ( 1.6) | 0.414 |
| Chemotherapy, yes, n (%) | 621 (58.7) | 306 (60.0) | 315 (57.5) | 0.442 |
| White blood cells (median (IQR)) | 5.90 (4.50, 7.82) | 6.72 (5.12, 9.09) | 5.22 (4.24, 6.60) | <0.001 |
| Neutrophil (median (IQR)) | 3.43 (2.26, 5.40) | 4.63 (2.97, 6.88) | 2.84 (1.90, 3.90) | <0.001 |
| Lymphocyte (median (IQR)) | 1.35 (0.97, 1.80) | 1.21 (0.90, 1.70) | 1.46 (1.02, 1.90) | <0.001 |
| Platelets (median (IQR)) | 216.50 (163.00, 278.00) | 225.50 (166.00, 291.75) | 209.00 (161.75, 266.00) | 0.006 |
| Red blood cells (median (IQR)) | 4.10 (3.63, 4.56) | 3.95 (3.46, 4.44) | 4.23 (3.78, 4.68) | <0.001 |
| Hemoglobin (median (IQR)) | 119.20 (101.00, 135.00) | 113.00 (95.00, 130.00) | 124.50 (107.00, 140.00) | <0.001 |
| Albumin (median (IQR)) | 38.65 (34.60, 42.10) | 35.50 (32.20, 38.98) | 41.00 (38.30, 43.90) | <0.001 |
| CRP | 3.64 (2.29, 19.28) | 20.76 (8.69, 53.22) | 2.42 (0.86, 3.20) | <0.001 |
| PGSGA score (median (IQR)) | 7.00 (4.00, 11.00) | 9.00 (6.00, 12.00) | 6.00 (3.00, 9.00) | <0.001 |
| Cachexia, yes, n (%) | 633 (59.8) | 405 (79.4) | 228 (41.6) | <0.001 |
| Status, death, n (%) | 453 (42.8) | 263 (51.6) | 190 (34.7) | <0.001 |
| Length of hospitalization (median (IQR)) | 9.00 (7.00, 14.00) | 11.00 (7.00, 15.00) | 9.00 (6.00, 12.00) | <0.001 |
| Hospitalization expenses(median (IQR)) | 22356.90 (12084.85, 57362.60) | 23542.00 (12333.43, 53665.55) | 20400.40 (11679.40, 62239.30) | 0.889 |

**Table S2.** The sensitivity analysis by excluding the first 3 months mortalities (58 patients).

| NCR | Model a | p value | Model b | p value | Model c | p value |
| --- | --- | --- | --- | --- | --- | --- |
| Continuous (per SD) | 0.819 (0.709,0.946) | 0.007 | 0.866 (0.748,1.002) | 0.053 | 0.901 (0.782,1.037) | 0.146 |
| Cutoff value |  |  |  |  |  |  |
| C1 (<178) | ref |  | ref |  | ref |  |
| C2 (≥178） | 0.577 (0.473,0.704) | <0.001 | 0.638 (0.521,0.78) | <0.001 | 0.699 (0.566,0.863) | 0.001 |
| Quartiles |  |  |  |  |  |  |
| Q1 (<44.49) | ref |  | ref |  | ref |  |
| Q2 (44.49-202.25) | 1.214 (0.937,1.573) | 0.142 | 1.006 (0.775,1.306) | 0.965 | 0.951 (0.729,1.241) | 0.714 |
| Q3 (202.25-430.46) | 0.824 (0.623,1.091) | 0.176 | 0.78 (0.589,1.033) | 0.083 | 0.811 (0.607,1.085) | 0.158 |
| Q4 (≥430.46) | 0.523 (0.391,0.699) | <0.001 | 0.544 (0.407,0.728) | <0.001 | 0.585 (0.433,0.789) | <0.001 |
| p for trend |  | <0.001 |  | <0.001 |  | <0.001 |

Notes:

Model a: No adjusted.

Model b: Adjusted for age, sex, TNM stage.

Model c: Adjusted for age, sex, TNM stage, surgery, radiotherapy, chemotherapy, hypertension, diabetes, smoking, drinking, family history.

**Table S3. Comparison of NCR with established inflammation-nutrition prognostic indices**

| Index | Components | Main biological dimension | Strengths | Limitations | Position relative to NCR |
| --- | --- | --- | --- | --- | --- |
| GPS | CRP and albumin | Systemic inflammation and hypoalbuminemia | Simple, widely used, clinically familiar | Categorical scoring may reduce granularity; does not include BMI | NCR retains CRP and albumin information while adding BMI-based nutritional reserve |
| mGPS | CRP-dominant score with albumin | Inflammation-dominant systemic response | Validated in many cancer settings; easy to apply | Strongly CRP-driven; limited assessment of body reserve | NCR may provide a broader inflammation-nutrition signal |
| PNI | Albumin and lymphocyte count | Nutritional and immune status | Simple and inexpensive; reflects immune-nutritional status | Does not directly include CRP or body size | NCR directly incorporates CRP and BMI, complementing PNI |
| NLR | Neutrophil-to-lymphocyte ratio | Inflammatory and immune balance | Easily available from routine blood counts | May be affected by infection, stress, or treatment; does not assess nutrition | NCR is less leukocyte-centered and more focused on nutrition-inflammation balance |
| SII | Platelet x neutrophil / lymphocyte | Systemic inflammation and immune activation | Captures multiple blood-cell components | Can fluctuate with infection, bleeding, thrombosis, or treatment | NCR may be easier to interpret as a nutritional-inflammatory marker |
| CAR | CRP-to-albumin ratio | Inflammation-nutrition imbalance | Closely related to inflammatory and albumin status | Does not include BMI or body reserve | NCR extends CAR-like information by including BMI |
| LCR | Lymphocyte-to-CRP ratio | Immune-inflammatory status | Reflects inflammation and immune suppression | Does not include albumin or BMI | NCR provides additional nutritional-reserve information |
| NCR | BMI x albumin / CRP | Integrated nutritional reserve and systemic inflammation | Uses routine clinical indicators; inexpensive; continuous; clinically accessible | Cohort-derived cutoff; modest standalone ROC performance; requires external validation | Should be considered a complementary risk-stratification marker rather than a replacement for existing indices |

Note: NCR should be interpreted as a complementary risk-stratification marker rather than a replacement for existing scores, because the present study did not perform a head-to-head comparison of all indices.
